# Analysis of epithelial–mesenchymal transition markers in psoriatic epidermal keratinocytes

**DOI:** 10.1098/rsob.150032

**Published:** 2015-08-12

**Authors:** Xiao-Yong Man, Xi-Bei Chen, Wei Li, Lilla Landeck, Ting-Ting Dou, Jia-qi Chen, Jiong Zhou, Sui-Qing Cai, Min Zheng

**Affiliations:** 1 Department of Dermatology, Second Affiliated Hospital, Zhejiang University School of Medicine, Hangzhou 310009, People's Republic of China; 2 Department of Dermatology, Ernst von Bergmann General Hospital, Teaching Hospital of Charité–University, Potsdam, Germany

**Keywords:** epithelial–mesenchymal transition, psoriasis, keratinocyte, E-cadherin, vimentin

## Abstract

Psoriasis is similar to endpoints of epithelial–mesenchymal transition (EMT), a process of epithelial cells transformed into fibroblast-like cells. The molecular epithelial and mesenchymal markers were analysed in psoriatic keratinocytes. No obvious alteration of epithelial markers E-cadherin (E-cad), keratin 10 (K10), K14 and K16 was detected in psoriatic keratinocytes. However, significantly increased expression of Vim, FN, plasminogen activator inhibitor 1 (PAI-1) and Slug was seen. IL-17A and IL-13 at 50 ng ml^−1^ strongly decreased expression of K10, Vim and FN. TGF-β1 at 50 ng ml^−1^ promoted the production of N-cad, Vim, FN and PAI-1. Slug was decreased by dexamethasone (Dex), but E-cad was upregulated by Dex. Silencing of ERK partially increased E-cad and K16, but remarkably inhibited K14, FN, Vim, β-catenin, Slug and *α*5 integrin. Moreover, inhibition of Rho and GSK3 by their inhibitors Y27632 and SB216763, respectively, strongly raised E-cad, β-catenin and Slug. Dex decreased Y27632-mediated increase of β-catenin. Dex at 2.0 µM inhibited SB216763-regulated E-cad, β-catenin and slug. In conclusion, EMT in psoriatic keratinocytes may be defined as an intermediate phenotype of type 2 EMT. ERK, Rho and GSK3 play active roles in the process of EMT in psoriatic keratinocytes.

## 1. Introduction

Psoriasis is clinically characterized by well-demarcated, erythematous plaques with adherent silvery scales [1]. The mitotic rate of the basal keratinocytes is dramatically increased: keratinocytes transit within 3–5 days from the basal to the cornified layer instead of 30 days in normal skin [2]. As a result, the epidermis is profoundly thickened (acanthosis), with elongated rete ridges. This is accompanied by an elongated spindle-shaped morphology in the upper stratum spinosum and an altered differentiation pattern of keratinocytes, indicated by parakeratosis, hyperkeratosis, loss of the granular layer as well as aberrant expression of various differentiation-associated antigens [1,2]. Furthermore, psoriatic keratinocytes are more resistant to apoptosis compared with normal keratinocytes [3]. These cellular processes, including loss of differentiation, increase in proliferation, resistance to apoptosis, and increase in migratory and invasive capacities, are similar to many endpoints of epithelial–mesenchymal transition (EMT) [4,5]. EMT is defined as epithelial cells losing their polarity and cohesiveness, and transforming into spindle-shaped cells that are more fibroblast- or myofibroblast-like [6–10].

The EMT is a key developmental programme that is often activated during cancer invasion and metastasis [7,11]. A defining characteristic of EMT is the loss of epithelial phenotypes and cell polarity, and the acquisition of a mesenchymal phenotype, including attenuation of E-cadherin (E-cad), ZO-1, cytokeratins, laminin 1 and microRNA-200 family, and acquisition of N-cadherin (N-cad), vimentin (Vim), β-catenin, fibronectin (FN), Snail, Slug and α-SMA, etc. [10,12–14].

We hypothesized that EMT may play a crucial role in psoriatic keratinocytes, and compared the expression and regulation of epithelial markers E-cad, keratin 10 (K10), K14 and K16, as well as mesenchymal markers Vim, FN, N-cad, β-catenin, plasminogen activator inhibitor 1 (PAI-1), Snail and Slug, in normal and psoriatic samples. According to our observations, it seems plausible that EMT at least superficially resembles the evolution from normal to transformed cell phenotype during psoriasis progression.

## 2. Material and methods

### 2.1. Reagents

Dispase, defined keratinocyte serum-free medium (KSFM) supplemented with keratinocyte growth factor (KGF), high glucose Dulbecco's modified Eagle's medium (DMEM) and fetal bovine serum (FBS) were obtained from Life Technologies (Gibco and Invitrogen, Auckland, CA, USA). SB-216763 and Y27632 were from Cayman Chemicals (Ann Arbor, MI, USA). IL-8, IL-13, IL-17A, TGF-β1and TNF-*α* were purchased from PeproTech (Rocky Hill, NJ, USA). 4′,6-diamidino-2-phenylindole (DAPI) was purchased from Sigma-Aldrich (St Louis, MO, USA). Fluorescein isothiocyanate (FITC)-conjugated, Cy3-conjugated and horseradish peroxidase (HRP)-conjugated secondary antibodies were purchased from Jackson ImmunoResearch Laboratories (West Grove, PA, USA). Cocktail protease inhibitors were purchased from Roche Diagnostics (Indianapolis, IN, USA). Primary antibodies used are listed in electronic supplementary material, table S1.

### 2.2. Human subjects

Skin biopsy samples were obtained as previously described [15]. A cohort of 41 psoriasis patients (mean age 45.7 ± 18.1, 15 females, 26 males) were included in the study. Patients did not receive any kind of psoriasis treatment for at least one month. A surgical biopsy of 1 cm untreated lesional skin from the arm or lower back was taken from the patients. Biopsies were also taken from 30 age-matched healthy volunteers (mean age 45.2 ± 17.3, 12 females, 18 males), who served as controls. The biopsies were used for immunohistochemical analysis and/or incubated in 0.5% dispase at 4°C overnight. Paraffin-embedded tissue blocks were available for each case.

### 2.3. Immunofluorescent and immunohistochemical analyses

Immunofluorescence was performed according to our previously published works [15–17]. Immunohistochemical analysis was performed using the standard ABC-peroxidase from Vector, using diaminobenzidine as the substrate (Vector, Burlingame, Ontario, CA, USA) as suggested by the manufacturer [18]. A panel of antibodies listed in electronic supplementary material, table S1 was used to define the expression of proteins. Affinity-purified biotinylated anti-rabbit or anti-mouse IgG was purchased from Vector Lab (Burlingame, Ontario, CA, USA). A representative picture showing similar results for each group was chosen for publication. Negative controls without primary antibodies showed no immunolabelling.

### 2.4. Primary keratinocyte culture and stimulation

Primary normal human keratinocytes (*n* = 16) and psoriatic lesional epidermal keratinocytes (*n* = 20) were established from skin biopsy samples incubated with dispase as earlier described [15–17]. Psoriatic epidermal keratinocytes were seeded onto 6-well plates. After 70% confluence, cells were starved for 24 h with basal K-SFM, and then treated with 50 ng ml^−1^ IL-17A, TNF-α, IL-8, TGF-β1 and 1 L-13. Furthermore, to determine the role of ERK, Rho and GSK3 in the process of EMT in psoriatic keratinocytes, cells were pretreated with inhibitors U0126, Y27632 and SB 216763 at 20 µM, separately for 2 h, then incubated with or without Dex at 0.2 µM and 2.0 µM for 24 h.

### 2.5. RNA sequencing

RNA was extracted from normal (*n* = 3) and psoriatic (*n* = 3) keratinocytes cultured *in vitro* by using TRIZol kit (Invitrogen, Carlsbad, CA, USA) according to the manufacturer's protocol. In addition, RNA from psoriatic lesional keratinocytes treated with or without IL-17A, TNFα, IL-8, TGF-β1 and IL-13 was also extracted. RNA sequencing was carried out by BGI (Shenzhen, China). Gene expression profile was assessed by RNAseq analysis. The gene expression was normalized to the number of reads per kilobase per million mapped reads (RPKM). The threshold for significant differentially expressed genes was judged based on FDR (false discovery rate) ≤ 0.001 and the absolute value of log_2_ratio ≥ 1 (fold change ≥ 2).

### 2.6. Quantitative real-time PCR

Total RNA was extracted from normal (*n* = 12) and psoriatic (*n* = 14) keratinocytes. Vim, FN, PAI-1, E-cad, N-cad, β-catenin, K10, K14, K16, Snail, Slug and β-actin mRNA were analysed by quantitative real-time PCR (qRT-PCR). The primers used for PCR were designed by Beacon Designer v. 8.0 (Premier Software) and are listed in the electronic supplementary material, table S2. A total of 2 µg RNA was reversed into cDNA by using Superscript II Reverse Transcriptase (Invitrogen). Gene expression was determined using SYBR green PCR mix (Roche) and 10 ng of template. Real-time PCR was performed on an ABI StepOne Plus instrument, using the following amplification conditions: 10 s at 95°C, followed by 40 cycles of 10 s at 95°C and 1 min at 60°C. CT values were analysed using qBase Plus 2 software (Biogazelle, Zwijnaarde, Belgium).

### 2.7. Western blot

Western blot was carried out as previously described [15]. Primary antibodies are listed in the electronic supplementary material, table S1. β-actin was measured as a loading control.

### 2.8. Statistical analysis

The statistical analyses were performed using SPSS software v. 18.0 (IBM SPSS Inc., Chicago, IL, USA) by means of one-way analysis of variance followed by the *t-*test. Grouped data are expressed as mean ± s.d.

## 3. Results

### 3.1. Immunohistochemical results of the epithelial and mesenchymal markers in normal and psoriatic skin

**Figure 1.**
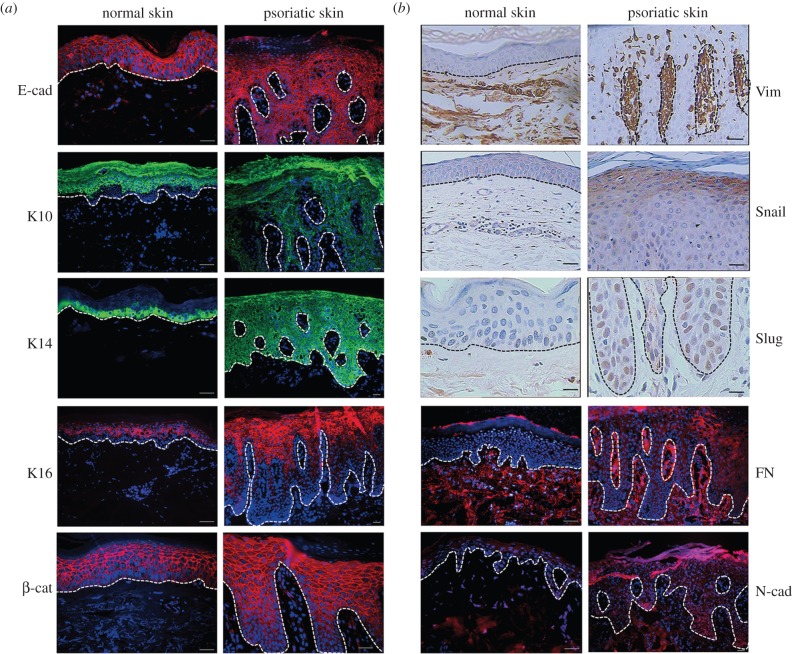
Immunohistochemical analysis of epithelial and mesenchymal markers in normal and psoriatic skin. Skin samples are collected by punch biopsy. Positive expression of (*a*) E-cad, K10, K14, K16, β-cat and (*b*) Vim, Snail, Slug, FN and N-cad are detected in normal and psoriatic epidermis. White or black dot lines represent the basal membrane. The images shown here are from a single representative experiment out of at least six repeats. Scale bar, 50 µm.

Immunolocalization of epithelial markers, including E-cad, K10, K14, K16 and mesenchymal markers, β-catenin, N-Cad, Vim, FN, Snail and Slug in normal and psoriatic skin were determined by immunohistochemistry. As shown in figure 1*a*, E-cad and β-catenin are distributed in the whole epidermal layer and are detected in cellular membrane, not in nuclei, in both normal and psoriatic epidermal keratinocytes. Cytoplasmic K10 and K16 localize in the middle and upper spinous epidermal keratinocytes and are absent in basal and suprabasal keratinocytes, in both normal and psoriatic epidermis. In normal epidermis, K14 localizes in the basal and suprabasal keratinocytes. However, in psoriatic epidermis, K14 is evenly distributed in the whole epidermal layers. No obvious difference of the above signals is detected between normal and psoriatic keratinocytes.

In normal and psoriatic dermis, little or no fluorescent signals of E-cad, K10, K14 and K16 are detected. On the contrary, signals of Vim, Snail, Slug, FN and N-cad are strong or obvious. Very faint or no signals of Vim, Snail, Slug, FN and N-cad are detected in normal epidermis (figure 1*b*). However, in the psoriatic epidermis, Vim, Snail, Slug and FN become strongly expressed (figure 1*b*). N-cad is moderately expressed. Furthermore, in psoriatic epidermal keratinocytes, Vim, Snail and FN are distributed in the cytoplasm. Slug is expressed in the nuclei. N-cad localizes in both cytoplasm and nuclei (figure 1*b*).

The immunohistochemical results of skin samples are listed in table 1.

**Table 1.** Immunohistochemical results of epidermal and mesenchymal markers in the epidermis and dermis of normal and psoriatic skin. +, positive; −, negative.

		epidermis		dermis	
		normal	psoriasis	normal	psoriasis
epidermal markers	E-cadherin	+	+	+/−	+/−
	K10	+	+	−	−
	K14	+	+	−	−
	K16	+	+	−	−
mesenchymal markers	vimentin	−	+	+	+
	Snail	−	+	+	+
	Slug	−	+	+	+
	fibronectin	−	+	+	+
	β-catenin	+	+	+	+
	N-cadherin	+/−	+	+	+

### 3.2. Expression of epithelial and mesenchymal markers in normal and psoriatic epidermal keratinocytes

**Figure 2.**
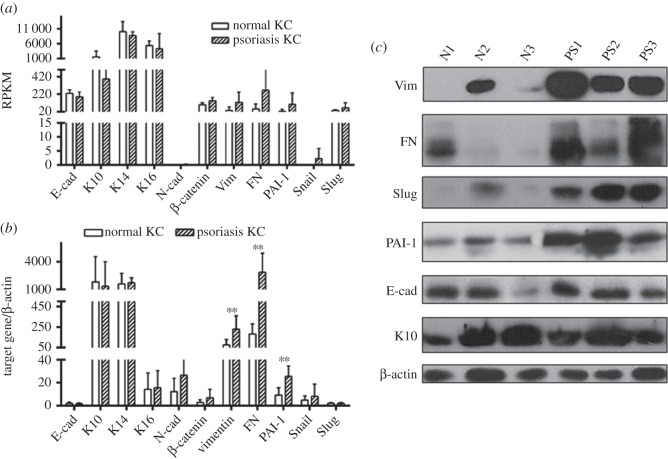
Expression of epithelial and mesenchymal markers at mRNA and protein levels in normal and psoriatic keratinocytes. RNA and protein were extracted from normal and psoriatic keratinocytes and cultured *in vitro* in defined KSFM. (*a*) RNAseq results. The expression of target genes is marked as RPKM. (*b*) qRT-PCR results. The expression of these markers is indicated by the ratio of the target gene to β-actin. Error bars are standard deviation between samples. **p < 0.01. (*c*) Comparative protein levels of Vim, FN, Slug, PAI-1, E-cad and K10 in normal and psoriatic keratinocytes determined by Western blot. Target protein expression was compared with β-actin expression.

The expression of epithelial and mesenchymal markers at the mRNA level was first determined by RNA sequencing in three normal and three psoriatic keratinocytes. As shown in figure 2*a*, expression of these markers is defined by RPKM. Decreased K10 and increased Vim, FN, PAI-1 and Snail are observed in psoriatic versus normal keratinocytes. No obvious differences in E-cad, K14, K16, N-cad, β-catenin and Slug are detected. To further verify the RNA sequencing data, an additional 12 normal and 14 psoriatic keratinocytes were included and analysed by qRT-PCR. As shown in figure 2*b*, there were significant differences of Vim, FN and PAI-1 in normal and psoriatic keratinocytes (*p* < 0.01). No statistically significant difference between the two groups was observed for E-cad, K10, K14, K16, N-cad, β-catenin, Snail and Slug.

We further contrasted protein levels of Vim, FN, Slug E-cad and K10 between normal and psoriatic keratinocytes. As shown in figure 2*c*, levels of Vim, FN and Slug were higher in psoriatic cells. No difference was seen for E-cad and K10. These results were in accordance with mRNA results, except for Slug, which showed increased protein levels without increase in mRNA.

### 3.3. Regulation of epithelial and mesenchymal markers in normal and psoriatic keratinocytes

**Figure 3.**
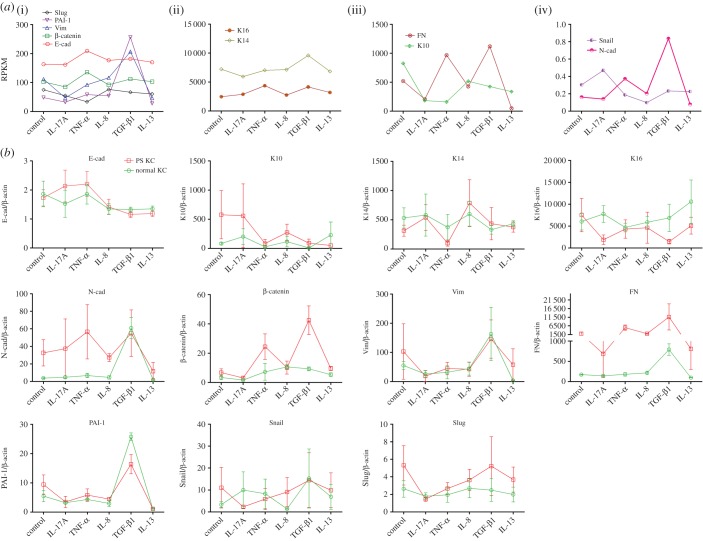
Regulation of epithelial and mesenchymal markers by IL-17A, TNF-α, IL-8, TGF-β1 and IL-13 at the RNA level. Psoriatic epidermal keratinocytes were starved for 24 h and then incubated with 50 ng ml^−1^ IL-17A, TNF-α, IL-8, TGF-β1 and IL-13 for 24 h. Then total RNA was extracted by the TRIZol kit. (*a*) The expression of target genes is indicated as RPKM determined by RNAseq and (*b*) the ratio of the target gene to β-actin determined by qRT-PCR.

To date, there are limited data in the literature on cytokines and signalling mechanisms regulating cutaneous EMT [19]. To determine the regulation of the expression of epithelial and mesenchymal markers, psoriatic keratinocytes were incubated with IL-17A, TNF-α, IL-8, TGF-β1 and IL-13 first, and then mRNA were determined by RNAseq and qRT-PCR analysis. As shown in figure 3*a*, E-cad is slightly increased by TNF-α. K10 was markedly reduced by IL-8, TGF-β1, IL-13, and especially IL-17A and TNF-α. K14 was downregulated by IL-17A, but upregulated by TGF-β1. K16 was evidently increased by TNF-α and TGF-β1. N-cad was remarkably increased by TGF-β1. Vim and FN was decreased by IL-17A and IL-13, but increased by TGF-β1. In addition, TNF-α upregulated FN, not Vim. PAI-1 was augmented by TGF-β1. Snail was increased by IL-17A but decreased by IL-8. Slug was markedly reduced by TNF-α. The detailed results are presented in table 2.

**Table 2.** Regulation of epidermal and mesenchymal markers by IL-17A, TNF-α, IL-8, TGF-β1 and IL-13. —, change less than 50%; ↑/↓, up/down at 50–100%; ↑↑/↓↓, up/down at 100–200%; ↑↑↑/↓↓↓, up/down more than 300%.

	IL-17A	TNF-α	IL-8	TGF-β1	IL-13
E-cad	—	—	—	—	—
K10	↓↓↓	↓↓↓	—	—	↓
K14	—	—	—	—	—
K16	—	↑↑	—	↑↑	—
N-cad	—	↑↑	—	↑↑↑	↓
β-catenin	—	—	—	—	—
Vim	↓↓	—	—	↑↑	↓↓
FN	↓↓	↑	—	↑↑	↓↓↓
PAI-1	—	—	—	↑↑↑	—
Snail	↑	—	↓	—	—
Slug	—	↓	—	—	—

The RNAseq results were further confirmed by qRT-PCR in normal and psoriatic keratinocytes (figure 3*b*). In psoriatic keratinocytes, most of the RNAseq results were repeated, except for K14 and K16. This discrepancy may be because of the heterogeneity of studied samples. More samples should be included.

### 3.4. The role of ERK, Rho and GSK3 in EMT in psoriatic keratinocytes

**Figure 4.**
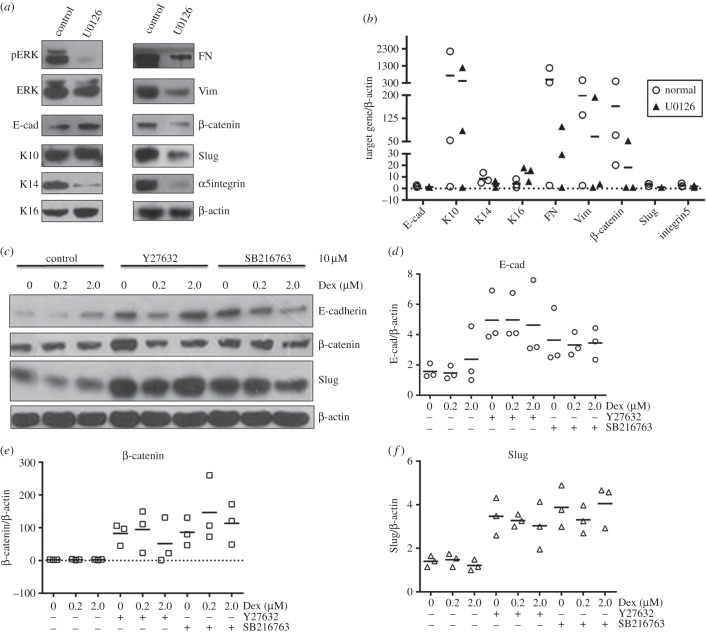
The role of ERK, Rho, GSK and Dex in the expression of epithelial and mesenchymal markers in psoriatic lesional keratinocytes. (*a*,*b*) Psoriatic keratinocytes were treated with U0126, and indicated target gene was determined by (*a*) Western blot (one representative example out of at least three similar repeats) and (*b*) qRT-PCR separately. (*c*–*f*) Psoriatic keratinocytes were pretreated with Y27632 or SB216763 for 2 h, then incubated with or without Dex at 0.2 or 2.0 µM. Indicated target gene was determined by Western blot and qRT-PCR separately. β-actin is used as loading control in (*a*) and (*c*). U0126, ERK inhibitor; Y27632, Rho inhibitor; SB216763, GSK3 inhibitor.

To further elucidate the role of ERK in EMT, an ERK inhibitor, U0126, was included to incubate with psoriatic keratinocytes. As shown in figure 4*a*, U0126 almost completely blocks the phosphorylation of ERK and increases E-cad and K16. However, U0126 decreases K14, FN, Vim, β-catenin, Slug and α5 integrin. No effect of U0126 on K10 was observed. These results were confirmed by further qRT-PCR, which show similar results as determined by Western blot (figure 4*b*).

The activation of Rho/ROCK signalling pathways are a key step in EMT-associated renal, lens, liver and bronchial epithelial cell fibrosis [20–23]. Glycogen synthase kinase 3 (GSK3) is a modulator of EMT [24]. Therefore, the specific inhibitor for Rho effector ROCK, Y27632, and inhibitor for GSK3 were used to inhibit the activity of Rho and GSK3, respectively; furthermore, dexamethasone (Dex) was added to check their role in EMT. Figure 4*c–f* shows that inhibition of Rho by Y27632 or inhibition of GSK3 increases the expression of E-cad, β-catenin and Slug at both protein and RNA levels. Dex at 0.2 µM decreases Slug but shows no effect on E-cad and β-catenin. However, Dex at 2.0 µM increases the expression of E-cad, but decreases Slug. Furthermore, Dex at 0.2 µM attenuates Y27632-mediated accumulation of E-cad and β-catenin, and Slug slightly. However, Dex at 2.0 µM does not change Y27632-mediated enhancement of these proteins, except for β-catenin, which is decreased. SB216763-mediated overexpression of E-cad is attenuated by Dex at both 0.2 and 2.0 µM. SB216763-mediated accumulation of β-catenin and Slug was not changed by Dex at 0.2 µM, but decreased by Dex at 2.0 µM.

## 4. Discussion

The mechanisms underlying EMT have been extensively explored in the past decade in cancer. Given the essentiality of the phenotypic changes in proliferation and migration of psoriatic epidermal keratinocytes, and accumulating evidence that suggests that psoriatic epidermal keratinocytes are resistant to apoptosis [3,25], we considered the possible involvement of EMT in the evolution of psoriatic epidermis. For this purpose, we examined the expression of epithelial and mesenchymal markers at the mRNA and protein level in normal and psoriasis patients.

One of the hallmarks of EMT is the loss of E-cad function [9,26]. Decreased expression of E-cad and β-catenin in active psoriatic lesional skin was earlier detected by immunohistochemical analysis [27,28]. Furthermore, increased nuclear β-catenin in suprabasal involved psoriatic epidermis was described by Hampton *et al.* [29]. In contrast to these results, we have not measured obvious changes either in the expression or in the distribution pattern of E-cad and β-catenin by immunohisochemistry, qRT-PCR, RNAseq and Western blot. Our results are in accordance with previous reports that there is no difference of E-cad between psoriatic and normal epidermis [30–32]. These inconsistent results may be because of the different laboratory methods being used. Immunohistochemistry alone is inadequate for quantitative analyses. It should be complemented whenever possible with quantitative measurements derived from other methods [33].

Epidermal keratinocytes express two major pairs of keratin polypeptides, K5/K14 and K1/K10. K5/K14 is expressed specifically in the basal generative compartment and K1/K10 specifically in the differentiating suprabasal compartment [34]. Previous reports have shown that psoriatic epidermis contains lower amounts of K10 and higher amounts of K14 and K16 [35,36]. In our study, no statistical differences for K10, K14 and K16 were detected between normal and psoriatic keratinocytes by using RNAseq, qRT-PCR and/or Western blot. These inconsistent results may be because of methods used in defining the expression of these proteins.

Mesenchymal markers, including Vim, FN, PAI-1, N-cad, Snail and Slug, were further investigated. Vim is used to track the proliferative state of melanocytes [37]. Percentages of Vim-positive cells correlate with erythema in psoriatic epidermal single cell suspensions determined by flow cytometric analysis [38]. In a proportion of biopsies of psoriatic lesions, PAI-1 was found to be present by immunohistochemistry [39,40]. Plasma levels of PAI-1 were significantly elevated in psoriatic patients and effectively diminished by treatment with a TNF-α inhibitor, but they were not reduced by UVB treatment [41,42]. Snail and Slug are transcription factors, and both are key mediators of EMT [43,44]. To our knowledge, there are no data on the expression of N-cad, Snail and Slug in psoriasis. Our study demonstrates the expression of Vim, FN, PAI-1, N-cad, Snail and Slug in psoriatic epidermal keratainocytes, determined by immunohistochemistry, RNAseq, qRT-PCR or Western blot at mRNA and protein levels. Moreover, Vim, FN, PAI-1 and Slug were significantly increased.

Therefore, there are increased mesenchymal markers and few differences in the expression of epithelial markers in psoriatic keratinocytes. Together with the morphological changes in psoriatic epidermis, such as spindle-like keratinocytes found in the upper stratum of spinous layer, remaining granular layer and stratum corneum, it is reasonable to conceive that psoriatic epidermal keratinocytes may experience a partial EMT.

There are three types of EMT [8]. Type 1 EMT gives rise to the mesoderm and endoderm, and to mobile neural crest cells. Type 2 EMT is associated with wound healing, tissue regeneration and organ fibrosis. Type 3 EMT is secondary epithelialization associated with cancer cells, and enables invasion and metastasis [8]. Psoriasis resembles tissue regeneration [45], and therefore EMT in psoriatic keratinocytes may be defined as type 2 EMT. Colocalization of these two sets of epithelial and mesenchymal markers defines an intermediate phenotype of EMT [8]. Our study has shown that both epithelial markers and mesenchymal markers are detected in psoriatic keratinocytes, which indicates that psoriatic epidermal keratinocytes have passed only partly through an EMT, and this transition is incomplete. Therefore, the EMT in psoriatic keratinocytes may be defined as an intermediate phenotype of type 2 EMT.

Next, we investigated the regulation of epithelial and mesenchymal markers in psoriatic keratinocytes. It is well known that TGF-β has been characterized as a paradigm for analyses of EMT [46,47]. Previous reports have shown that IL-17A, TNF-α or IL-8 alone or synergized with TGF-β1 promotes EMT in different cells [48–53]. IL-13 promotes the expression of genes associated with cell invasion in HT29 intestinal epithelial cells [54]. Hence, the roles of these cytokines were conducted in psoriatic keratinocytes. IL-17A strongly decreases expression of K10, Vim and FN. TNF-α clearly downregulates K10 and Slug but upregulates K16 and FN. Evidently, TGF-β1 promotes the production of N-cad, Vim, FN and PAI-1. In addition, IL-13 decreases K10, Vim and especially FN. No obvious effect of IL-8 was observed, except for the decrease of Snail. Thus, one may conclude that IL-17A and IL-13 are negative regulators, whereas TGF-β1 is an enhancer for EMT in psoriatic keratinocytes. The effect of TNF-α may be pluripotent; additional data should be obtained to establish its role. These results are partly in accordance with the published data [48–54]. Herein, the role of cytokines in regulating EMT may be determined not only by their actions; the unique characteristics of the cells are also critical.

TGF-β induces EMT through its TGF-β receptors and the activation of the Smad signalling pathway, as well as non-Smad signalling cascades including MAPK/ERK, Rho-family of GTPases and PI3 K/Akt [55,56]. Our findings show that silencing of ERK partially increases E-cad and K16, but remarkably inhibits FN, Vim, β-catenin, Slug and α5 integrin. Therefore, ERK plays an active role in the process of EMT. In line with our observations, other authors have shown that the ERK pathway is required for EMT and contributes to the maintenance of an undifferentiated/mesenchymal state in tumour cells [57–59].

Topical glucocorticoid is one of the most commonly used anti-inflammatory drugs in psoriasis [15]. Dex plays a protective role in preventing EMT in HK-2 cells and human peritoneal mesothelial cells [60,61], by inhibiting GSK-3β phosphorylation and by Snail upregulation [61]. In addition, activation of the Rho/ROCK pathway in Schlemm's canal endothelial cells contributes to the mechanism of Dex-induced changes [62]. Dex may also play a protective role in EMT in psoriatic keratinocytes partly through inducing E-cad and inhibiting Slug protein. On the other hand, suppression of Rho and GSK3 strongly increases E-cad, β-catenin and Slug. It seems that there is an inverse correlation between the activity of Rho and GSK3, and expression of Slug and E-cad at the protein level in psoriatic keratinocytes. The identical increase of epithelial and mesenchymal markers by the above-mentioned inhibitors is paradoxical, as Slug can repress E-cadherin transcription [63]. As previously reported, inhibition of Rho by Y27632 upregulated the E-cad expression in silica-stimulated bronchial epithelial cells [23], but decreased Slug promoter activity in human ovarian cancer cells [64]. The inverse correlation between the GSK3 activity and Slug protein levels was also observed in a lung adenocarcinoma cell line (CL1–5). Activation of GSK3β made a conspicuous decrease in Slug protein levels [65]. GSK3 may in turn regulate the expression of Slug [65]. However, John *et al.* have reported that GSK3β inhibition is associated with the decreased expression of Slug and N-cadherin in melanoma [66]. These discrepancies may lead to the possibility that Rho and GSK3 may act depending on the cell type. Meanwhile, compared with the controls, Dex does not change Y27632-mediated enhancement of E-cad and Slug, but decreases Y27632-mediated increase of β-catenin and further suppresses Y27632-mediated decrease of K14. High dosage of Dex (2.0 µM) inhibits SB216763-regulated E-cad, β-catenin and Slug. Therefore, Dex may regulate the levels of some proteins through the Rho and GSK3 pathways.

In summary, our investigations provide support for defining EMT in psoriatic epidermal keratinocytes as an intermediate phenotype of type 2 EMT characterized by upregulation of Vim, FN and PAI-1, with no changes in the expression of E-cad, K10 and K14. In the EMT process in psoriatic keratinocytes, IL-17A, IL-13 and TGF-β1 are important regulators, and ERK, Rho and GSK3 play important roles. Additionally, Dex plays a protective role. Although the detailed mechanistic role remains largely unknown, the current study clearly highlights the potential target for clinical treatment of psoriasis. For better understanding of molecular processes of EMT in psoriasis, additional regulatory factors have to be studied in the future.

## Supplementary Material

Supplementary Materials

## References

[1] Nestle FO, Kaplan DH, Barker J 2009 Psoriasis. N. Engl. J. Med. 361, 496–509. (doi:10.1056/NEJMra0804595) 19641206 10.1056/NEJMra0804595

[2] Schon MP, Boehncke WH 2005 Psoriasis. N. Engl. J. Med. 352, 1899–1912. (doi:10.1056/NEJMra041320) 15872205 10.1056/NEJMra041320

[3] Wrone-Smith T, Mitra RS, Thompson CB, Jasty R, Castle VP, Nickoloff BJ 1997 Keratinocytes derived from psoriatic plaques are resistant to apoptosis compared with normal skin. Am. J. Pathol. 151, 1321–1329. 9358758 PMC1858068

[4] Sleeman JP 2006 Complex networks orchestrate epithelial–mesenchymal transitions. Nat. Rev. Mol. Cell Biol. 7, 131–142. (doi:10.1038/nrm1835) 10.1038/nrm1835 16493418

[5] Said NA, Williams ED 2011 Growth factors in induction of epithelial–mesenchymal transition and metastasis. Cells Tissues Organs 193, 85–97. (doi:10.1159/000320360) 21051862 10.1159/000320360

[6] Kalluri R, Neilson EG 2003 Epithelial–mesenchymal transition and its implications for fibrosis. J. Clin. Invest. 112, 1776–1784. (doi:10.1172/JCI20530) 14679171 10.1172/JCI20530 PMC297008

[7] Hugo H, Ackland ML, Blick T, Lawrence MG, Clements JA, Williams ED, Thompson EW 2007 Epithelial–mesenchymal and mesenchymal–epithelial transitions in carcinoma progression. J. Cell Physiol. 213, 374–383. (doi:10.1002/jcp.21223) 10.1002/jcp.21223 17680632

[8] Kalluri R, Weinberg RA 2009 The basics of epithelial–mesenchymal transition. J. Clin. Invest. 119, 1420–1428. (doi:10.1172/JCI39104) 19487818 10.1172/JCI39104 PMC2689101

[9] Acloque H, Adams MS, Fishwick K, Bronner-Fraser M, Nieto MA 2009 Epithelial–mesenchymal transitions: the importance of changing cell state in development and disease. J. Clin. Invest. 119, 1438–1449. (doi:10.1172/JCI38019) 10.1172/JCI38019 PMC2689100 19487820

[10] Zeisberg M, Neilson EG 2009 Biomarkers for epithelial–mesenchymal transitions. J. Clin. Invest. 119, 1429–1437. (doi:10.1172/JCI36183) 19487819 10.1172/JCI36183 PMC2689132

[11] Mani SA et al. 2008 The epithelial–mesenchymal transition generates cells with properties of stem cells. Cell 133, 704–715. (doi:10.1016/j.cell.2008.03.027) 18485877 10.1016/j.cell.2008.03.027 PMC2728032

[12] Christiansen JJ, Rajasekaran AK 2006 Reassessing epithelial to mesenchymal transition as a prerequisite for carcinoma invasion and metastasis. Cancer Res. 66, 8319–8326. (doi:10.1158/0008-5472.CAN-06-0410) 16951136 10.1158/0008-5472.CAN-06-0410

[13] Vasko V et al. 2007 Gene expression and functional evidence of epithelial-to-mesenchymal transition in papillary thyroid carcinoma invasion. Proc. Natl Acad. Sci. USA 104, 2803–2808. (doi:10.1073/pnas.0610733104) 17296934 10.1073/pnas.0610733104 PMC1815262

[14] Schmalhofer O, Brabletz S, Brabletz T 2009 E-cadherin, beta-catenin, and ZEB1 in malignant progression of cancer. Cancer Metastasis Rev. 28, 151–166. (doi:10.1007/s10555-008-9179-y) 19153669 10.1007/s10555-008-9179-y

[15] Man XY et al. 2013 Impaired nuclear translocation of glucocorticoid receptors: novel findings from psoriatic epidermal keratinocytes. Cell Mol. Life Sci. 70, 2205–2220. (doi:10.1007/s00018-012-1255-3) 23334186 10.1007/s00018-012-1255-3 PMC11113139

[16] Man XY, Yang XH, Cai SQ, Bu ZY, Zheng M 2008 Overexpression of vascular endothelial growth factor (VEGF) receptors on keratinocytes in psoriasis: regulated by calcium independent of VEGF. J. Cell Mol. Med. 12, 649–660. (doi:10.1111/j.1582-4934.2007.00112.x) 18419602 10.1111/j.1582-4934.2007.00112.x PMC3822550

[17] Man XY, Yang XH, Cai SQ, Yao YG, Zheng M 2006 Immunolocalization and expression of vascular endothelial growth factor receptors (VEGFRs) and neuropilins (NRPs) on keratinocytes in human epidermis. Mol. Med. 12, 127–136. (doi:10.2119/2006-00024.Man) 17088944 10.2119/2006-00024.Man PMC1626599

[18] Man XY, Finnson KW, Baron M, Philip A 2012 CD109, a TGF-beta co-receptor, attenuates extracellular matrix production in scleroderma skin fibroblasts. Arthritis Res. Ther. 14, R144 (doi:0.1186/ar3877) 22694813 10.1186/ar3877 PMC3446527

[19] O'Kane D et al. 2014 SMAD inhibition attenuates epithelial to mesenchymal transition by primary keratinocytes *in vitro*. Exp. Dermatol. 23, 497–503. (doi:10.1111/exd.12452) 24848428 10.1111/exd.12452

[20] Patel S, Takagi KI, Suzuki J, Imaizumi A, Kimura T, Mason RM, Kamimura T, Zhang Z 2005 RhoGTPase activation is a key step in renal epithelial mesenchymal transdifferentiation. J. Am. Soc. Nephrol. 16, 1977–1984. (doi:10.1681/ASN.2004110943) 15901767 10.1681/ASN.2004110943

[21] Cho HJ, Yoo J 2007 Rho activation is required for transforming growth factor-beta-induced epithelial–mesenchymal transition in lens epithelial cells. Cell Biol. Int. 31, 1225–1230. (doi:10.1016/j.cellbi.2007.04.006) 17537651 10.1016/j.cellbi.2007.04.006

[22] Kitamura K et al. 2007 Rho/Rho kinase is a key enzyme system involved in the angiotensin II signaling pathway of liver fibrosis and steatosis. J. Gastroenterol. Hepatol. 22, 2022–2033. (doi:10.1111/j.1440-1746.2006.04735.x) 17914985 10.1111/j.1440-1746.2006.04735.x

[23] Hu YB, Li X, Liang GN, Deng ZH, Jiang HY, Zhou JH 2013 Roles of Rho/Rock signaling pathway in silica-induced epithelial–mesenchymal transition in human bronchial epithelial cells. Biomed. Environ. Sci. 26, 571–576. (doi:10.3967/0895-3988.2013.07.008) 23895702 10.3967/0895-3988.2013.07.008

[24] Doble BW, Woodgett JR 2007 Role of glycogen synthase kinase-3 in cell fate and epithelial–mesenchymal transitions. Cells Tissues Organs 185, 73–84. (doi:10.1159/000101306) 17587811 10.1159/000101306

[25] Weatherhead SC, Farr PM, Jamieson D, Hallinan JS, Lloyd JJ, Wipat A, Reynolds NJ 2011 Keratinocyte apoptosis in epidermal remodeling and clearance of psoriasis induced by UV radiation. J. Invest. Dermatol. 131, 1916–1926. (doi:10.1038/jid.2011.134) 21614017 10.1038/jid.2011.134 PMC3160491

[26] Thiery JP, Acloque H, Huang RY, Nieto MA 2009 Epithelial–mesenchymal transitions in development and disease. Cell 139, 871–890. (doi:10.1016/j.cell.2009.11.007) 19945376 10.1016/j.cell.2009.11.007

[27] Li Z, Peng Z, Wang Y, Geng S, Ji F 2008 Decreased expression of E-cadherin and beta-catenin in the lesional skin of patients with active psoriasis. Int. J. Dermatol. 47, 207–209. (doi:10.1111/j.1365-4632.2007.03318.x) 18211502 10.1111/j.1365-4632.2007.03318.x

[28] Chung E, Cook PW, Parkos CA, Park YK, Pittelkow MR, Coffey RJ 2005 Amphiregulin causes functional downregulation of adherens junctions in psoriasis. J. Invest. Dermatol. 124, 1134–1140. (doi:10.1111/j.0022-202X.2005.23762.x) 15955087 10.1111/j.0022-202X.2005.23762.x

[29] Hampton PJ, Ross OK, Reynolds NJ 2007 Increased nuclear beta-catenin in suprabasal involved psoriatic epidermis. Br. J. Dermatol. 157, 1168–1177. (doi:10.1111/j.1365-2133.2007.08195.x) 17916213 10.1111/j.1365-2133.2007.08195.x

[30] Zhou S, Matsuyoshi N, Takeuchi T, Ohtsuki Y, Miyachi Y 2003 Reciprocal altered expression of T-cadherin and P-cadherin in psoriasis vulgaris. Br. J. Dermatol. 149, 268–273. 12932231 10.1046/j.1365-2133.2003.05464.x

[31] Lemini-Lopez A, Flores-Romo L, Arevalo-Lopez A, Meza I 2006 Altered morphology and distribution of cellular junction proteins in non-lesional psoriatic epidermis: an insight into disease severity. Arch. Med. Res. 37, 36–44. (doi:10.1016/j.arcmed.2005.07.003) 16314184 10.1016/j.arcmed.2005.07.003

[32] Donetti E, Gualerzi A, Ricceri F, Pescitelli L, Bedoni M, Prignano F 2012 Etanercept restores a differentiated keratinocyte phenotype in psoriatic human skin: a morphological study. Exp. Dermatol. 21, 549–551. (doi:10.1111/j.1600-0625.2012.01518.x) 22716254 10.1111/j.1600-0625.2012.01518.x

[33] Fritschy JM 2008 Is my antibody-staining specific? How to deal with pitfalls of immunohistochemistry. Eur. J. Neurosci. 28, 2365–2370. (doi:10.1111/j.1460-9568.2008.06552.x) 19087167 10.1111/j.1460-9568.2008.06552.x

[34] Rao KS, Babu KK, Gupta PD 1996 Keratins and skin disorders. Cell Biol. Int. 20, 261–274. 8664850

[35] Bernard BA, Asselineau D, Schaffar-Deshayes L, Darmon MY 1988 Abnormal sequence of expression of differentiation markers in psoriatic epidermis: inversion of two steps in the differentiation program? J. Invest. Dermatol. 90, 801–805. 3286778 10.1111/1523-1747.ep12462014

[36] Thewes M, Stadler R, Korge B, Mischke D 1991 Normal psoriatic epidermis expression of hyperproliferation-associated keratins. Arch. Dermatol. Res. 283, 465–471. (doi:10.1007/Bf00371784) 1724897 10.1007/BF00371784

[37] Batacsorgo Z, Hammerberg C, Voorhees JJ, Cooper KD 1993 Flow cytometric identification of proliferative subpopulations within normal human epidermis and the localization of the primary hyperproliferative population in psoriasis. J. Exp. Med. 178, 1271–1281. (doi:10.1084/jem.178.4.1271) 7690831 10.1084/jem.178.4.1271 PMC2191196

[38] Glade CP, VanErp PEJ, VandeKerkhof PCM 1996 Epidermal cell DNA content and intermediate filaments keratin 10 and vimentin after treatment of psoriasis with calcipotriol cream once daily, twice daily and in combination with clobetasone 17-butyrate cream or betamethasone 17-valerate cream: a comparative flow cytometric study. Br. J. Dermatol. 135, 379–384. 8949429

[39] Gissler HM, Frank R, Kramer MD 1993 Immunohistochemical characterization of the plasminogen activator system in psoriatic epidermis. Br. J. Dermatol. 128, 612–618. 7687853 10.1111/j.1365-2133.1993.tb00254.x

[40] Lyons-Giordano B, Loskutoff D, Chen CS, Lazarus G, Keeton M, Jensen PJ 1994 Expression of plasminogen activator inhibitor type 2 in normal and psoriatic epidermis. Histochemistry 101, 105–112. 8071082 10.1007/BF00269356

[41] Nielsen HJ, Christensen IJ, Svendsen MN, Hansen U, Werther K, Brunner N, Petersen LJ, Kristensen JK 2002 Elevated plasma levels of vascular endothelial growth factor and plasminogen activator inhibitor-1 decrease during improvement of psoriasis. Inflamm. Res. 51, 563–567. 12540021 10.1007/pl00012428

[42] Sigurdardottir G, Ekman AK, Stahle M, Bivik C, Enerback C 2014 Systemic treatment and narrowband ultraviolet B differentially affect cardiovascular risk markers in psoriasis. J. Am. Acad. Dermatol. 70, 1067–1075. (doi:10.1016/j.jaad.2013.12.044) 24656729 10.1016/j.jaad.2013.12.044

[43] Nieto MA 2002 The snail superfamily of zinc-finger transcription factors. Nat. Rev. Mol. Cell Biol. 3, 155–166. (doi:10.1038/nrm757) 11994736 10.1038/nrm757

[44] Barrallo-Gimeno A, Nieto MA 2009 Evolutionary history of the Snail/Scratch superfamily. Trends Genet. 25, 248–252. (doi:10.1016/j.tig.2009.04.001) 19427053 10.1016/j.tig.2009.04.001

[45] Nickoloff BJ, Bonish BK, Marble DJ, Schriedel KA, DiPietro LA, Gordon KB, Lingen MW 2006 Lessons learned from psoriatic plaques concerning mechanisms of tissue repair, remodeling, and inflammation. J. Investig. Dermatol. Symp. Proc. 11, 16–29. 10.1038/sj.jidsymp.5650010 17069007

[46] Derynck R, Muthusamy BP, Saeteurn KY 2014 Signaling pathway cooperation in TGF-beta-induced epithelial–mesenchymal transition. Curr. Opin. Cell Biol. 31C, 56–66. (doi:10.1016/j.ceb.2014.09.001) 25240174 10.1016/j.ceb.2014.09.001 PMC4657734

[47] Katsuno Y, Lamouille S, Derynck R 2013 TGF-beta signaling and epithelial–mesenchymal transition in cancer progression. Curr. Opin. Oncol. 25, 76–84. (doi:10.1097/CCO.0b013e32835b6371) 23197193 10.1097/CCO.0b013e32835b6371

[48] Vittal R et al. 2013 IL-17 induces type V collagen overexpression and EMT via TGF-beta-dependent pathways in obliterative bronchiolitis. Am. J. Physiol. Lung Cell Mol. Physiol. 304, L401–L414. (doi:10.1152/ajplung.00080.2012) 23262228 10.1152/ajplung.00080.2012 PMC3602743

[49] Ji X et al. 2013 IL4 and IL-17A provide a Th2/Th17-polarized inflammatory milieu in favor of TGF-beta1 to induce bronchial epithelial–mesenchymal transition (EMT). Int. J. Clin. Exp. Pathol. 6, 1481–1492. 23923066 PMC3726963

[50] Techasen A, Namwat N, Loilome W, Duangkumpha K, Puapairoj A, Saya H, Yongvanit P 2014 Tumor necrosis factor-alpha modulates epithelial mesenchymal transition mediators ZEB2 and S100A4 to promote cholangiocarcinoma progression. J. Hepatobiliary Pancreat. Sci. 21, 703–711. (doi:10.1002/jhbp.125) 24867797 10.1002/jhbp.125

[51] Bates RC, Mercurio AM 2003 Tumor necrosis factor-alpha stimulates the epithelial-to-mesenchymal transition of human colonic organoids. Mol. Biol. Cell. 14, 1790–1800. (doi:10.1091/mbc.E02-09-0583) 12802055 10.1091/mbc.E02-09-0583 PMC165077

[52] Yu JP et al. 2013 Dysfunctional activation of neurotensin/IL-8 pathway in hepatocellular carcinoma is associated with increased inflammatory response in microenvironment, more epithelial mesenchymal transition in cancer and worse prognosis in patients. PLoS ONE 8, e56069 (doi:10.1371/journal.pone.0056069) 23418512 10.1371/journal.pone.0056069 PMC3572009

[53] Yin J, Zeng F, Wu N, Kang K, Yang Z, Yang H 2015 Interleukin-8 promotes human ovarian cancer cell migration by epithelial–mesenchymal transition induction in vitro. Clin. Transl. Oncol. 17, 365–370. (doi:10.1007/s12094-014-1240-4) 25373532 10.1007/s12094-014-1240-4

[54] Scharl M et al. 2013 Interleukin-13 and transforming growth factor beta synergise in the pathogenesis of human intestinal fistulae. Gut 62, 63–72. (doi:10.1136/gutjnl-2011-300498) 22287592 10.1136/gutjnl-2011-300498

[55] Valcourt U, Kowanetz M, Niimi H, Heldin CH, Moustakas A 2005 TGF-β and the Smad signaling pathway support transcriptomic reprogramming during epithelial-mesenchymal cell transition. Mol. Biol. Cell 16, 1987–2002. (doi:10.1091/mbc.E04-08-0658) 15689496 10.1091/mbc.E04-08-0658 PMC1073677

[56] Derynck R, Zhang YE 2003 Smad-dependent and Smad-independent pathways in TGF-beta family signalling. Nature 425, 577–584. (doi:10.1038/nature02006) 14534577 10.1038/nature02006

[57] Maurer G, Tarkowski B, Baccarini M 2011 Raf kinases in cancer-roles and therapeutic opportunities. Oncogene 30, 3477–3488. (doi:10.1038/onc.2011.160) 21577205 10.1038/onc.2011.160

[58] Ma B, Wells A 2014 The mitogen-activated protein (MAP) kinases p38 and extracellular signal-regulated kinase (ERK) are involved in hepatocyte-mediated phenotypic switching in prostate cancer cells. J. Biol. Chem. 289, 11 153–11 161. (doi:10.1074/jbc.M113.540237) 10.1074/jbc.M113.540237 PMC4036254 24619413

[59] Neuzillet C, Tijeras-Raballand A, de Mestier L, Cros J, Faivre S, Raymond E 2014 MEK in cancer and cancer therapy. Pharmacol. Ther. 141, 160–171. (doi:10.1016/j.pharmthera.2013.10.001) 24121058 10.1016/j.pharmthera.2013.10.001

[60] Li Q, Lv LL, Wu M, Zhang XL, Liu H, Liu BC 2013 Dexamethasone prevents monocyte-induced tubular epithelial–mesenchymal transition in HK-2 cells. J. Cell Biochem. 114, 632–638. (doi:10.1002/jcb.24405) 23060286 10.1002/jcb.24405

[61] Jang YH et al. 2013 Effects of dexamethasone on the TGF-beta1-induced epithelial-to-mesenchymal transition in human peritoneal mesothelial cells. Lab. Invest. 93, 194–206. (doi:10.1038/labinvest.2012.166) 23207448 10.1038/labinvest.2012.166

[62] Fujimoto T, Inoue T, Kameda T, Kasaoka N, Inoue-Mochita M, Tsuboi N, Tanihara H 2012 Involvement of RhoA/Rho-associated kinase signal transduction pathway in dexamethasone-induced alterations in aqueous outflow. Invest. Ophthalmol. Vis. Sci. 53, 7097–7108. (doi:10.1167/iovs.12-9989) 22969074 10.1167/iovs.12-9989

[63] Bolos V, Peinado H, Perez-Moreno MA, Fraga MF, Esteller M, Cano A 2003 The transcription factor Slug represses E-cadherin expression and induces epithelial to mesenchymal transitions: a comparison with Snail and E47 repressors. J. Cell Sci. 116, 499–511. (doi:10.1242/Jcs.00224) 12508111 10.1242/jcs.00224

[64] Peng J, Zhang G, Wang QS, Huang JG, Ma H, Zhong YH, Zhou FX, Xie CH, Zhang A 2012 ROCK cooperated with ET-1 to induce epithelial to mesenchymal transition through SLUG in human ovarian cancer cells. Biosci. Biotechnol. Biochem. 76, 42–47. (doi:10.1271/Bbb.110411) 22232246 10.1271/bbb.110411

[65] Kao SH et al. 2014 GSK3beta controls epithelial–mesenchymal transition and tumor metastasis by CHIP-mediated degradation of Slug. Oncogene 33, 3172–3182. (doi:10.1038/onc.2013.279) 23851495 10.1038/onc.2013.279 PMC4096338

[66] John JK et al. 2012 GSK3beta inhibition blocks melanoma cell/host interactions by downregulating N-cadherin expression and decreasing FAK phosphorylation. J. Invest. Dermatol. 132, 2818–2827. (doi:10.1038/jid.2012.237) 22810307 10.1038/jid.2012.237 PMC3479306

