# Supplementary material for: Analysis of epithelial–mesenchymal transition markers in psoriatic epidermal keratinocytes
Source: Open Biol. 2015 Aug 12;5(8):150032. doi: 10.1098/rsob.150032 (PMC4554915; doi:10.1098/rsob.150032)
Supplement: Supplementary Materials [file rsob150032supp1.pdf]

Supplementary Materials

Supplementary Table 1. Antibodies used in the study

Antibodies	Type	Catalogue number	Source
E-cadherin	R	SC-7870	SCBT
K10	M	SC-51581	SCBT
K14	M	SC-53253	SCBT
K16	M	SC-53255	SCBT
Erk1/2	R	9102	CST
pErk1/2	R	9106S	CST
Vimentin	M	SC-73259	SCBT
Fibronectin	R	SC-9068	SCBT
PAI-1	R	SC-8979	SCBT
N-cadherin	R	SC-7939	SCBT
Snail	R	3879S	CST
Slug	R	9585S	CST
â-catenin	R	SC-7199	SCBT
Integrin α5	M	SC-136224	SCBT
â-actin	M	SC-47778	SCBT

M, mouse monoclonal; R, rabbit polyclonal;

SCBT Santa Cruz Biotech; CST, Cell Signal Technology

Supplementary Table 2. Primers for EMT markers

Accession Number	Gene	Sense Primer	Anti-sense Primer
NM_004360	E-cadherin	ACATACACTCTCTTCTCTC	GTCATTCTGATCGGTTAC
NM_000421	K10	CAGATTCTCAACCTAACA	TCATTCTCATACTTCAGC
NM_000526	K14	AGGAACAAGATTCTCACA	TTCAACTCTGTCTCATACT
NM_005557	K16	GAGGAACAAGATCATTGC	TCATACTTGGTCCTGAAG
NM_003380	Vimentin	AACCTGAGGGAAACTAAT	TTGATAACCTGTCCATCT
NM_212482	Fibronectin	AACCTGAAGCTGAAGAGA	TAGGACGCTCATAAGTGT
NM_000602	PAI-1	TAGAGAACCTGGGAATGAC	GAGGCTCTTGGTCTGAAA
NM_001792	N-cadherin	ATCATCCTGCTTATCCTT	TTATCTCTTACATCATCTTCTG
NM_001904	β-catenin	AAGCCACAAGATTACAAGAA	ATCAGCAGTCTCATTCCA
NM_005985	Snail	CGCTCTTTCCTCGTCAGG	TGGAAGGTAAACTCTGGATTAGA
NM_003068	Slug	GACACACATACAGTGATTATTTC	CTGAGGATCTCTGGTTGT
NM_001101	*β*-actin	GATGACCCAGATCATGTTTG	CGTACAGGGATAGCACAG
